# GOLD standard dataset for Alzheimer genes

**DOI:** 10.1016/j.dib.2020.105439

**Published:** 2020-04-01

**Authors:** Sushrutha Raj, Anchal Vishnoi, Alok Srivastava

**Affiliations:** a Amity Institute of Integrative Sciences and Health, Amity University Haryana, Amity Education Valley, Gurgaon 122413, India; b Institute of Bioinformatics and Computational Biology, Visakhapatnam, Andhra Pradesh 530017, India

**Keywords:** Alzheimer genes, Cross validation, GOLD standard, Meta analysis, System modeling, Text classification, Machine learning, Alzheimer gene association

## Abstract

Alzheimer disease is a genetically complex multigenic neurodegenerative disorder, resulting from the interaction between multiple genes. Most of the earlier studies reported only few specific genes that have involvement in Alzheimer. However more than hundreds of susceptible genes have been observed, that have significant role in the development and progression of Alzheimer. Among all the existing data resources, Genetic association database is the most popular data source that contains information about genes, their association classes into positive, negative and neutral class and supporting reference. However, it contains lot of false positives and negatives associations. We have taken this data as reference and performed the double fold cross validation to compile the comprehensive list of Alzheimer genes, their association class viz, positive, negative or ambiguous with the disease and reference sentence confirming the association. The data generated will be used as a GOLD standard reference data set for the training of machine learning classifier to predict the classification of published literature not only in Alzheimer but in other diseases as well. In addition, positive associated genes data can also be used for the system level modelling or meta analysis of Alzheimer.

**Specifications table**

**Table:** 

Subject	Health Informatics
Specific subject area	Alzheimer genes, Alzheimer gene classification, Text classification
Type of data	Tables
How data were acquired	Raw data was downloaded from Genetic Association Database via https://geneticassociationdb.nih.gov/
Data format	Raw, Processed, and Analyzed
Parameters for data collection	Raw data collected contains disease name, disease class, gene, PubMed id, association class, title of article, year of publication and reference sentence supports the association.
Description of data collection	Raw data collected from an archives of published genetic association studies that contains information on molecular and clinical parameters.
Data source location	Institution: Amity Institute of Integrative Sciences and Health, Amity University Gurgaon. City/Region: Gurgaon/ Haryana Country: India
Data accessibility	Data are available on Mendeley data source via http://dx.doi.org/10.17632/643r5r2wrh.1

## Value of the Data

• The data presented herein would be the first open-access data useful as a GOLD standard reference data for the training of machine learning classifier to predict the classification of published literature not only in Alzheimer, but in other disease as well. This classified data can be further explored to map the genes with the classified document into positive, negative, or ambiguous class association, to study disease gene association. These associations are supported by weight criteria based on the empirical probability for each association class, that signifies the strength of association. This will help researchers to find the association class of Alzheimer related genes with a certain weight.
• This data would be helpful for the scientists and researchers working in the system level modelling of Alzheimer and also for investigators working in the domain of natural language processing and machine learning of text data, specially scientific literature data.
• This data would be useful in gene prioritization for Alzheimer, which in turn will be helpful in a variety of applications that ranges from DNA screening for early diagnosis, to gene mutation analysis and more effective drug development. Processed data also contains the ambiguous association of genes with the Alzheimer. There are 128 such genes, which can be used further to plan experiments to confirm their association with the disease.
• Positive associated Alzheimer genes can also be used for the system level modelling, meta analysis of disease or to study differential evolution of genes impact the disease.

## 1 Data description

The raw data collected in this study is described in raw data description, which is filtered to remove the redundant entries and further classified in different association classes. This raw data has been double folded cross validated to remove the false predictions, to generate the processed file. This processed file is used to calculate the weight of each gene association class. Details of each of them are discussed below.

### 1.1 Raw data description

We have used Genetic Association Database (GAD) [1] as a reference database to extract the information of Alzheimer related genes. GAD is a open access database of genetic association data of complex disease and disorder hosted by National Institute of Health. The data reported in GAD contains 89 columns, including the references and association class of genes with the disease. This association data is distributed into three classes, namely “Yes”, signifies the association of genes with disease, “No”, no association between disease and genes and “Not Defined”, whose relation to disease has not been determined. We have used the last freezed version of GAD to extract Alzheimer related information. The keywords “Alzheimer”, “alzheimer” and “ALZHEIMER” have been used to filter raw data and found 4047 records. Summary of Alzheimer data records are mentioned in Table 1.

**Table 1:** Summary of Alzheimer data downloaded from GAD. Unique classes represents the association class of the data.

Entities	Number
Duration	1984 - 2012
Unique number of genes	1338
Unique classes reported	3
Unique PUBMED ID	1900
No of entries	4047

For the purpose of our study, we filtered 8 columns from the raw data mentioned in the Table 2. Filtered raw data is included in Supplementary file 1.

**Table 2:** Column description of GAD data set filtered for Alzheimer query. *Association mentioned is described in three classes as “Yes”, “No” or “Not Defined”.

Column name	Description
DISEASE	Disease name
DIS_CLASS	Disease class
GENE	Gene Symbol
PUBMED ID	PubMed Id of the article
LACKASSO	Type of association*
TITLE	Title of the article
YEAR	Year of publication
CONCLUSION	Reference sentence

### 1.2 Filtering of raw data

We have processed Supplementary file 1 that contains 4047 records of the raw data mentioned in Table 1. We cleaned the data to remove the redundant entries, details of which are mentioned in Table 3.

**Table 3:** Summary of redundant entries removed from raw data to clean the data.

Category	Column Processed	Count
Duplicated entries	All	4
PMID Not Available	PUBMED ID	99
Genes not available	Gene	17
“variant(s)” as Gene Symbol	Gene	1
“NA” as Gene Symbol	Gene	1
“intergenic” and its variants as Gene Symbol	Gene	7
Non protein coding gene	Gene	61
Records needs to validate	–	3857
**Total**		**4047**

After cleaning Supplementary file 1, there were 3857 records available, which were further processed. Table 4, contains the summary of 3857 raw entries classified into different association classes and number of associated genes. Genes included in these classes are overlapped with multiple association classes.

**Table 4:** Summary of GAD classification into various association classes.

GAD's classification		
LACKASSO	No of Entries	No of genes
Y	551	209
N	762	517
B	2544	716
Total	3857	1442
Unique genes		1206

## 2 Experimental design, materials, and methods

Filtered records on 3857 entries have further been processed to create GOLD standard dataset.

### 2.1 Double fold cross validation

The above mentioned dataset have been double fold cross validated to remove the false positives (FP) and false negatives (FN) from the raw data. FP records are those which are wrongly reported as positive association with the disease, while FN are those having positive association on validation, and originally reported as not associated with disease. The reference sentence of raw data mentioned in CONCLUSION column is validated with respect to the association class mentioned in LACASSO column and mentioned gene in GENE column. If the association class information for a given gene is correctly referenced, we have kept it as it is otherwise we have modified the relevant reference from the same abstract. Incase reference does not have the clear association between the disease and gene, we classified it into several other sub categories mentioned in ASSOCIATION.CLASS as described in Table 5.

**Table 5:** Sub categories description of ASSOCIATION.CLASS of the processed data.

S No	Sub category	Description	Count
1	Y	Gene is positively associated with Alzheimer disease	642
2	N	Gene is not associated with Alzheimer disease	464
3	A	Gene is ambiguous in nature. Word “may” or “may not” is used to define the association of genes with Alzheimer disease.	254
4	MC	Mis Classified. Multiple entries for which PMID, gene symbol, reference sentences are same, but classification are different.	28
5	ANF	Abstract Not Found. For the given PMID abstract is not found in the reference article.	18
6	D	Duplicate entries. For multiple references genes, reference sentences, PMID and association class is same.	86
7	NR	Not Related. Abstract is not related to Alzheimer	134
8	PNF	PMID Not Found	12
9	X	Abstract does not contain any information about the association between mentioned gene in GENE column and Alzheimer.	2219
	**Total**		**3857**

### 2.2 Gene symbol conversion

Gene validated in the reference sentence is mentioned in terms of gene symbol, gene name, gene synonymous or its aliases. This gene information is presented in REF.GENE column. This information is further mapped to the approved gene symbol from HUGO Gene Nomenclature Committee (HGNC) [2] and updated as the approved gene symbol in the GENE.NEW column of the processed data.

### 2.3 Weight calculation

Approved gene symbol reported in GENE.NEW column is used to define the gene block, which may have multiple class associations. This data will be used to calculate the empirical probability for each association in a gene block. This gives the probability of association class of a gene to the disease.

In the final processed data, we have introduced 6 new columns as mentioned in Table 6.

**Table 6:** Description of columns added in raw data file.

S No	Column Label	Description
1	S.NO	Serial number
2	REF.SENTENCE	Validated reference sentence mentioned for disease gene association
3	ASSOCIATION.CLASS	Association class of validated reference sentence
4	REF.GENE	Gene symbol mentioned in the reference sentence. It could be genes symbol, gene name, aliases or synonymous terms for the referred gene.
5	GENE.NEW	Approved gene symbol of REF_GENE column, used to calculate WEIGHT
6	WEIGHT	Disease gene association probability

### 2.4 Gene class assignment

Once the probability is calculated, the class associated to a gene will be calculated by using maximum weight criteria, i.e., a gene is assigned to a particular association class based on the maximum weight obtained. Alternatively for the selection of positively associated genes, one can choose all genes annotated to positive class using the weight criteria. Processed data are included in Supplementary file 2.

The data prepared above contains double fold cross validated information on genes, their association class with disease and supporting reference. This can be used as a GOLD standard data set to design machine learning based classification algorithm to predict the class of the bio-medical abstracts available. Positive genes selected here can be used for system level meta analysis of Alzheimer.

## Appendix Supplementary materials

Image, application 1
